# Investigating the microvascular dysfunction in the Frontotemporal Dementia using a progranulin‐deficient mice model

**DOI:** 10.1002/alz.093422

**Published:** 2025-01-03

**Authors:** Supriya Chakraborty, Sofia Franciosa, Oliver Bracko

**Affiliations:** ^1^ University of Miami, Miami, FL USA; ^2^ University of Miami, Coral Gables, FL USA

## Abstract

**Background:**

Despite being the second most common form of dementia, vascular contributions of Frontotemporal Dementia are understudied. Recent data from patients and preliminary experiments have indicated that in progranulin‐deficient mice, an increased number of cortical capillaries are stalled, and cerebral blood flow is reduced. Here, we examined the underlying mechanism contributing to microvascular dysfunction in FTD.

**Method:**

Blood‐brain barrier (BBB) integrity was examined using immunohistochemistry and protein analyses. We compared PGRN‐deficient mice to controls and tested for the tight junction proteins Claudin‐5, Occludin, and markers of BBB leakage, such as Albumin. High Resolution in vivo two‐photon Imaging was utilized to detect the capillary stalls and capillary blood flow in the cortical capillaries, and the images were analyzed using MATLAB and ImageJ software.

**Result:**

We discovered that the number of blood vessels significantly decreased in the 10‐11 month‐old PGRN‐deficient mice compared to controls. Furthermore, the tight junction protein levels of Claudin‐5 and Occludin are significantly reduced in PGRN‐deficient mice compared to age and sex‐matched controls. Finally, in‐vivo multiphoton experiments revealed a reduction in cerebral blood flow associated with a increased number of capillary stalling.

**Conclusion:**

This study shows that the BBB is damaged in PGRN‐deficient mice, accompanied by microvascular dysfunction and hypoperfusion. The data suggest that PGRN‐deficient mice could be an ideal model to investigate vascular contributions to FTD.

